# Programmed BRD9 Degradation and Hedgehog Signaling Activation via Silk‐Based Core‐Shell Microneedles Promote Diabetic Wound Healing

**DOI:** 10.1002/advs.202404130

**Published:** 2024-10-16

**Authors:** Yili Liu, Mingliang Zhou, Jinrui Sun, Enhui Yao, Jingyi Xu, Guangzheng Yang, Xiaolin Wu, Ling Xu, Jiahui Du, Xinquan Jiang

**Affiliations:** ^1^ Department of Prosthodontics Shanghai Ninth People's Hospital Shanghai Jiao Tong University School of Medicine College of Stomatology Shanghai Jiao Tong University National Center for Stomatology National Clinical Research Center for Oral Diseases Shanghai Key Laboratory of Stomatology Shanghai Research Institute of Stomatology Shanghai Engineering Research Center of Advanced Dental Technology and Materials Shanghai 200125 China

**Keywords:** diabetes, Hedgehog signaling, microneedle patch, silk fibroin nanofibers, wound healing

## Abstract

Wound healing impairment in diabetes mellitus is associated with an excessive inflammatory response and defective regeneration capability with suppressed Hedgehog (Hh) signaling. The bromodomain protein BRD9, a subunit of the non‐canonical BAF chromatin‐remodeling complex, is critical for macrophage inflammatory response. However, whether the epigenetic drug BRD9 degrader can attenuate the sustained inflammatory state of wounds in diabetes remains unclear. Without a bona fide immune microenvironment, Hh signaling activation fails to effectively rescue the suppressed proliferative ability of dermal fibroblasts and the vascularization of endothelial cells. Therefore, a silk‐based core‐shell microneedle (MN) patch is proposed to dynamically modulate the wound immune microenvironment and the regeneration process. Specifically, the BRD9 degrader released from the shell of the MNs mitigated the excessive inflammatory response in the early phase. Subsequently, the positively charged Hh signaling agonist is released from the negatively charged core of the silk fibroin nanofibers and promotes the phase transition from inflammation to regeneration, including re‐epithelialization, collagen deposition, and angiogenesis. These findings suggest that the programmed silk‐based core‐shell MN patch is an ideal therapeutic strategy for effective skin regeneration in diabetic wounds.

## Graphical abstract

A programmed silk‐based core‐shell microneedle patch is developed for effective diabetic wound‐healing by sequentially modulating the immune microenvironment and wound regeneration process. Specifically, the BRD9 degrader released from the shell mitigates the excessive inflammatory response in the early phase. Subsequently, the positively charged Hedgehog signaling agonist released from the core promotes phase transition from inflammation to regeneration.

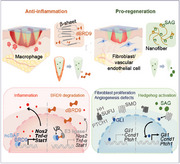

## 1 Introduction

Diabetes mellitus (DM) is one of the largest global health issue, with more than 10% of the world's population predicted to suffer from diabetes and its complications by 2021.^[^ ^1^, ^2^ ^]^ A particularly tough clinical problem for patients with diabetes is impaired wound healing, as ≈15–25% of them face a lifetime risk of developing diabetic foot ulcers, leading to significant morbidity and mortality worldwide.^[^ ^3^ ^]^ However, in‐depth knowledge of the cellular and molecular pathogenesis of delayed wound healing in DM and effective clinical therapeutic treatments is still lacking.^[^ ^4^, ^5^ ^]^

The pathophysiology of poor wound healing in patients with DM is complex and multifactorial.^[^ ^6^ ^]^ Previous studies revealed a link between impaired wound healing in DM and persistent chronic inflammation. Persistent chronic inflammation is associated with the hindered transition of macrophages to an anti‐inflammatory phenotype, resulting in a local increase in the levels of inflammatory cytokines such as interleukin‐1*β* (IL‐1*β*) and tumor necrosis factor‐*α* (TNF‐*α*).^[^ ^7^, ^8^ ^]^ Several recent studies have reported that epigenetic regulations in macrophages contribute to their sustained inflammatory state in DM, including DNA modification and the biochemical modification of histone tails.^[^ ^9^, ^10^, ^11^ ^]^ ATP‐dependent chromatin‐remodeling complexes regulate DNA accessibility via ATP hydrolysis‐mediated nucleosome mobilization and repositioning.^[^ ^12^ ^]^ The mammalian BAF family is an ATP‐dependent chromatin‐remodeling complex consisting of distinct subcomplexes.^[^ ^13^, ^14^ ^]^ The bromodomain protein BRD9 is a unique subunit of the non‐canonical BAF (ncBAF) complex. Previous studies have shown that BRD9 inhibitors and degraders inhibit inflammatory response of macrophages by specifically blocking BRD9‐mediated induction of interferon‐stimulated gene expression.^[^ ^15^, ^16^, ^17^, ^18^ ^]^ However, whether local BRD9 inhibition could attenuate the sustained inflammatory state of wounds in patients with diabetes remains to be elucidated.

In addition to sustained chronic inflammation, impaired keratinocyte and fibroblast function, and decreased secretion of growth factors, disruptions in the vascularization of endothelial cells in the later phases also impair wound healing ability in patients with DM.^[^ ^19^ ^]^ It has been shown that cutaneous injury‐induced Hedgehog (Hh) signaling activation during wound repair contributes to dermal fibroblast proliferation, microvascular remodeling, and hair follicle regeneration.^[^ ^20^ ^]^ Mouse models have shown that the Hh signaling pathway is compromised in diabetic skin.^[^ ^21^ ^]^ Therefore, by targeting the pathogenesis of delayed wound healing in DM, we hypothesized that the combination of a BRD9 degrader in the early inflammatory state and an Hh signaling agonist (smoothened agonist, SAG) in the later regeneration phase could be an ideal therapy for effective skin regeneration treatment in DM.

The key to implementing the aforementioned multidrug combination therapy is the construction of a differential drug release system. The development of microneedle (MN) technology, particularly the design of core‐shell structured MNs, has provided a new approach to repairing diabetic skin defects. MNs have been developed as a minimally invasive and effective approach for multifunctional transdermal delivery, penetrating the skin to form microchannels and enabling targeted drug delivery. This significantly improves drug utilization efficiency and serves as a promising alternative to traditional oral and injectable methods of drug delivery. Physical intervention with MNs could provide a low‐stress microenvironment for scar‐free wound healing by reducing contraction and mechanical stress in fibroblasts.^[^ ^22^ ^]^ In recent years, dissolvable MNs made from biocompatible and biodegradable materials have emerged as promising drug delivery systems.^[^ ^23^ ^]^ However, the materials available for constructing core‐shell‐structured MNs are relatively underdeveloped, limiting their potential for clinical applications.

Silk fibroin (SF), a natural biopolymer derived from silkworm cocoons, is an ideal material for preparing dissolvable MNs. It is well known for its excellent biocompatibility, controllable biodegradability, and adjustable mechanical properties.^[^ ^24^, ^25^ ^]^ The processing conditions for silk fibroin are mild, which preserves the stability and bioactivity of sensitive biological molecules and cells over extended periods.^[^ ^26^ ^]^ Moreover, silk‐based MNs have been widely used for cardiovascular and gastrointestinal wound healing owing to their versatility and efficacy.^[^ ^27^, ^28^ ^]^ These properties suggest that silk fibroin is an ideal candidate for developing drug‐loaded MNs specifically targeting diabetic skin wounds.

In this study, we developed a pure silk fibroin core‐shell MN fabrication strategy to dynamically modulate the wound immune microenvironment and the wound regeneration process, matching the healing phases in DM. Specifically, the shells of the MNs were equipped with the enhanced mechanical strength and toughness of silk fibroin by inducing *β*‐sheet formation using polyols. The BRD9 degrader loaded into the shell was rapidly released to modulate the local inflammatory environment during the early phase. The core employed the electronegativity and rapid gelation capabilities of silk fibroin nanofibers (SNFs). The negatively charged inner SNF not only retards the sustained release rate via physical obstruction but also reduces the release of positively charged SAG through electrostatic interactions. The released SAG promoted the transition from inflammation to proliferation, re‐epithelialization, collagen deposition, and angiogenesis. Collectively, our findings suggest that the programmed silk‐based core‐shell MN patch we developed is an ideal therapeutic strategy for effective skin regeneration in DM.

## 2 Results

### 2.1 Excessive Inflammation and Inhibited Dermal Regeneration in Diabetic Wounds

To explore an effective and targeted skin regeneration strategy for delayed wound healing in DM, we investigated the cellular mechanisms resulting in impaired skin regeneration in diabetic wounds.

**Figure 1:**
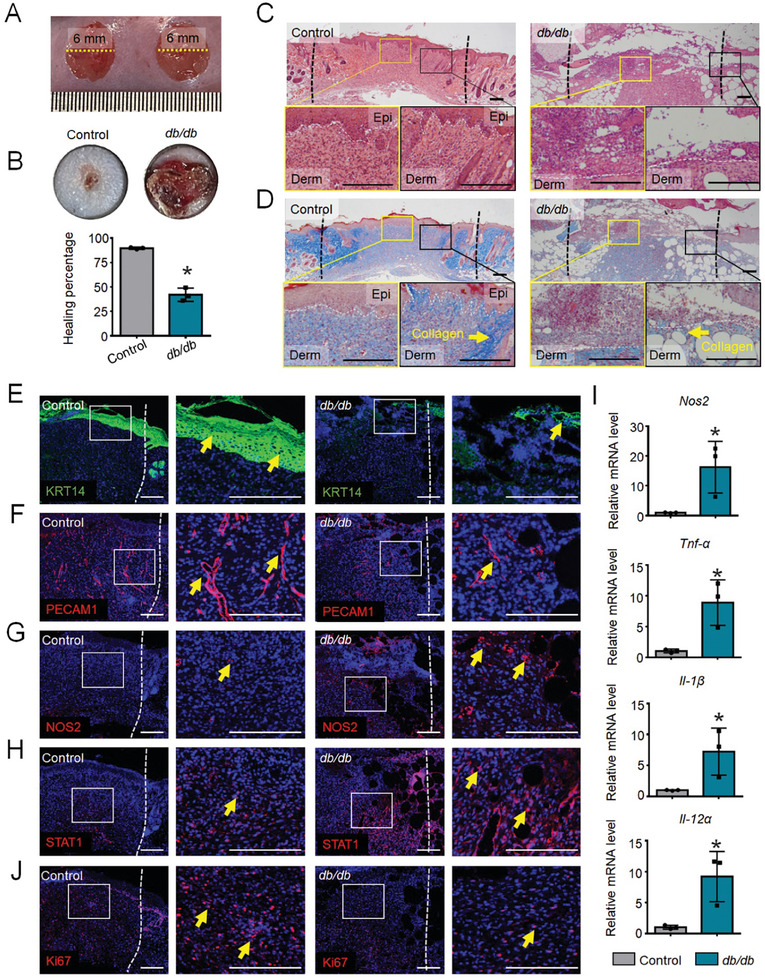
Excessive inflammation and inhibited dermal regeneration in diabetic wound healing. A) Representative image of the mouse dorsal wound healing model. B) Representative image and quantification of wound healing expressed as the % of wound closure in wild‐type (WT) and *db/db* mice 9 days after surgery. Data presented as the mean ± SD, n = 3. P‐values were calculated using a two‐tailed Student's t‐test. *P < 0.05. C) Representative H&E and D) Masson's trichrome staining images of wound healing in WT and *db/db* mice 9 days after surgery. The yellow arrow indicates the collagen bundles. Epi, epidermis; derm, dermis. Immunofluorescence staining for E) KRT14 (green), F) PECAM1 (red), G) NOS2 (red), H) STAT1 (red), and J) Ki67 (red) in the wounds of WT and *db/db* mice 9 days after surgery. The yellow arrows indicate positive signals. I) The mRNA expression of *Nos2, Tnf‐α, Il‐1β, and Il‐12α* in the wounds of WT and *db/db* mice 3 days after surgery, as measured by qPCR. Data are presented as the mean ± SD, n = 3. P‐values were calculated using two‐tailed Student's t‐test, *P < 0.05. Scale bar, 200 µm.

Type 2 DM affects a vast majority of patients with diabetes worldwide.^[^ ^29^ ^]^ In this study, we constructed a 6‐mm full‐thickness wound defect on the back skin of 8‐week‐old *BKS‐Lepr^em2Cd479/Nju^* (*Lepr^db/db^*, *db/db*) and age‐matched control mice. A representative image of the mouse dorsal wound‐healing model is shown in **Figure** **1**A. Wound areas were quantified and compared between control and *db/db* mice using ImageJ software, with photos taken 0 and 9 days after surgery (Figure 1B). Using hematoxylin and eosin (H&E) staining, we detected intact re‐epithelialization and dermal regeneration in the skin defects of control mice, indicating a good healing effect, but not in diabetic wounds (Figure 1C). To evaluate changes in collagen deposition in diabetic wounds, we performed Masson's trichrome staining. The results showed that collagen content increased during wound healing in wild‐type (WT) mice, whereas collagen deposition was severely compromised in *db/db* mice (Figure 1D). In addition, immunofluorescence analysis for keratin 14 (KRT14) (Figure 1E) and platelet/endothelial cell adhesion molecule 1 (PECAM1) (Figure 1F) in the wound tissue was performed. We found that re‐epithelialization and blood vessel formation at the wound site were both severely impaired in diabetic wounds. These results indicate that compromised wound healing in DM is associated with impaired keratinocyte and fibroblast function and a disruption in the vascularization of endothelial cells.

To further understand the pathogenesis of delayed wound healing in DM, we explored the underlying cellular and molecular mechanisms. We detected sustained chronic inflammation with disrupted macrophage function in DM via immunofluorescence staining for nitric oxide synthase 2 (NOS2) (Figure 1G) and signal transducer and activator of transcription 1 (STAT1) (Figure 1H). We further validated that the mRNA expression of inflammatory factors *Nos2*, *Tnf‐α, Il‐1β*, and *Il‐12α*, were significantly higher in the wounds of DM mice than those in control mice (Figure 1I). Furthermore, we found that the proliferation of dermal fibroblasts was impeded within the wounds of diabetic mice via immunofluorescence staining for Ki67, a marker for cell proliferation (Figure 1J).

### 2.2 BRD9 Degrader‐Loaded MN Patch Mitigates Excessive Inflammation in Diabetic Wound Healing

Previous studies reported that during macrophage activation, BRD9 cooperates with BRD4 and regulates the expression of interferon‐stimulated genes (ISGs); and BRD9 modulates the glucocorticoid receptor‐mediated repression of inflammatory genes.^[^ ^15^, ^16^ ^]^ The chemical PROTAC (proteolysis targeting chimera) dBRD9 is a targeted BRD9 degrader molecule that triggers BRD9 ubiquitination and proteasomal degradation, showing clinical therapeutic potential.^[^ ^15^, ^16^, ^17^, ^30^, ^31^, ^32^ ^]^ For example, dBRD9 treatment before Lipid A stimulation reduced ISG induction through reprogramming the chromatin binding profile of glucocorticoid receptors and enhancing their repression of inflammatory responses.^[^ ^15^, ^16^ ^]^ Our recent study also demonstrated that dBRD9 exerts preventive and therapeutic effects on acute periodontitis and alveolar bone loss.^[^ ^17^ ^]^

**Figure 2:**
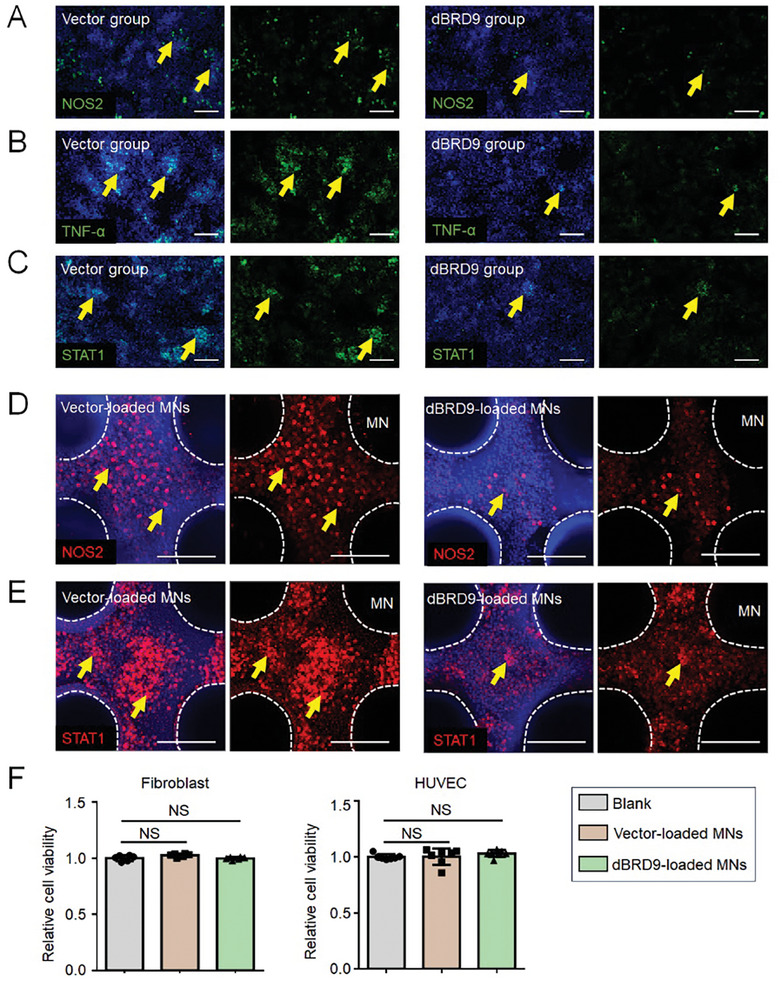
Anti‐inflammatory effects and biocompatibility of BRD9 degrader‐loaded MNs in vitro. Immunofluorescence staining for A) NOS2 (green), B) TNF‐*α* (green), and C) STAT1 (green) in dBRD9‐ or control vector‐treated macrophage RAW 264.7 cells after LPS stimulation. Yellow arrows indicate positive signals. Immunofluorescence staining for D) NOS2 (red) and E) STAT1 (red) in RAW 264.7 macrophage cells on dBRD9‐ or control vector‐loaded MNs under LPS stimulation. F) Cell viability of dermal fibroblasts and HUVECs cultured in the extracted solutions from control vector‐loaded MNs and the dBRD9‐loaded MNs for 24 h. A blank solution served as the control. Data are presented as the mean ± SD. P‐values were calculated using ANOVA with Dunnett's multiple comparisons test. For fibroblasts, n = 6. For HUVECs, n = 7. NS, not significant, P >0.05. Scale bar, 200 µm.

Therefore, we investigated whether the local application of dBRD9 could attenuate the sustained inflammatory state of diabetic wounds by epigenetically inhibiting macrophage polarization and activation. We initially evaluated the anti‐inflammation effect of BRD9 degradation in vitro. We found that dBRD9 could effectively alleviate macrophage polarization and inhibit the expression of NOS2, TNF‐*α*, and STAT1 (**Figure** **2**A–C).

To explore the effective application of dBRD9 in wound healing in a diabetic mouse model, silk‐based MN patches were fabricated for controlled‐release drug delivery as previously described.^[^ ^33^ ^]^ We seeded lipopolysaccharide (LPS)‐stimulated RAW 264.7 macrophages on the MN patch. We found that dBRD9‐loaded MNs significantly inhibited macrophage activation and reduced the expression levels of the inflammatory factors NOS2 and STAT1 compared to control vector‐loaded MNs (Figure 2D,E).

In addition to their anti‐inflammatory function, it is important to ensure that dBRD9‐loaded MNs are non‐cytotoxic and have no detrimental effects on dermal fibroblasts and endothelial cells. MN patches weighing 80 mg were soaked in 4 mL of complete culture medium for 48 h before the medium was collected for cytotoxicity evaluation. The viability of dermal fibroblasts and human umbilical vein endothelial cells (HUVECs) was assessed using the Cell Counting Kit‐8 (CCK8) assay, after cultured in solutions extracted from control vector‐loaded MNs and dBRD9‐loaded MNs for 24 h; a blank solution was used as the negative control. The results showed that dBRD9‐loaded and vector‐loaded MNs showed no apparent inhibition of cell viability compared to the blank control after 24 h of incubation (Figure 2F).

**Figure 3:**
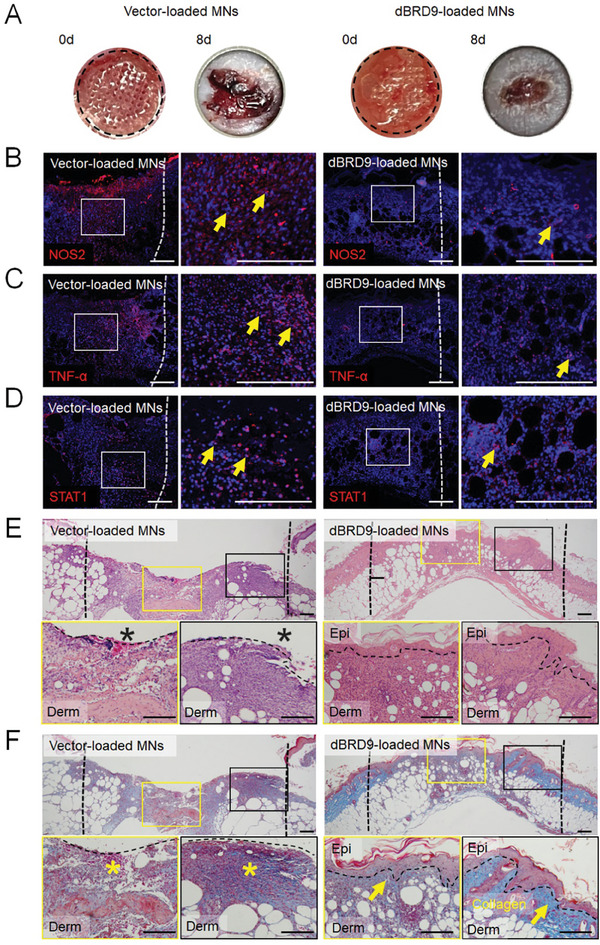
BRD9 degrader‐loaded MNs mitigate excessive inflammation in diabetic wound healing. A) Representative image of the wound healing of dBRD9‐loaded MNs and the control group in *db/db* mice at day 0 and day 8 after surgery. Immunofluorescence staining for B) NOS2 (red), C) TNF‐*α* (red), and D) STAT1 (red) in the wounds of the dBRD9‐loaded MNs group compared with the control group in *db/db* mice 8 days after surgery. Yellow arrows indicate positive signals. E) Representative H&E and F) Masson's staining images of wound healing in the dBRD9‐loaded MNs group and the control group of *db/db* mice 8 days after surgery. The yellow arrows indicate the collagen bundles. The asterisk indicates compromised re‐epithelialization in e and defective collagen deposition in f. Epi, epidermis; derm, dermis. Scale bar, 200 µm.

To assess the anti‐inflammatory function of the MNs on diabetic wound healing in a mouse model in vivo, we applied a dBRD9‐loaded MN patch to the skin wounds of diabetic mice; a vector‐loaded MN patch was used as a control. Assessment of wound healing coverage revealed that the dBRD9‐loaded MN patch promoted re‐epithelialization, indicating a better healing effect than the blank‐loaded MN patch (**Figure** **3**A). We also detected that sustained chronic inflammation with excessive macrophage polarization in DM was alleviated in the dBRD9‐loaded MN patch group, along with decreased expression of NOS2 (Figure 3B), TNF‐*α* (Figure 3C), and STAT1 (Figure 3D) compared with the control group. To further analyze the wound healing situation, we conducted the H & E and Masson's staining. We detected improved re‐epithelialization and collagen deposition in the skin defects in the dBRD9‐loaded MN patch group compared to the control group (Figure 3E,F).

### 2.3 Hh Signaling Downregulation Impairs Dermal Regeneration in DM

In addition to sustained chronic inflammation with excessive macrophage polarization, impeding dermal fibroblast proliferation and vascularization, primarily or after excessive inflammation, further contributes to impaired dermal regeneration ability in DM.^[^ ^19^ ^]^

**Figure 4:**
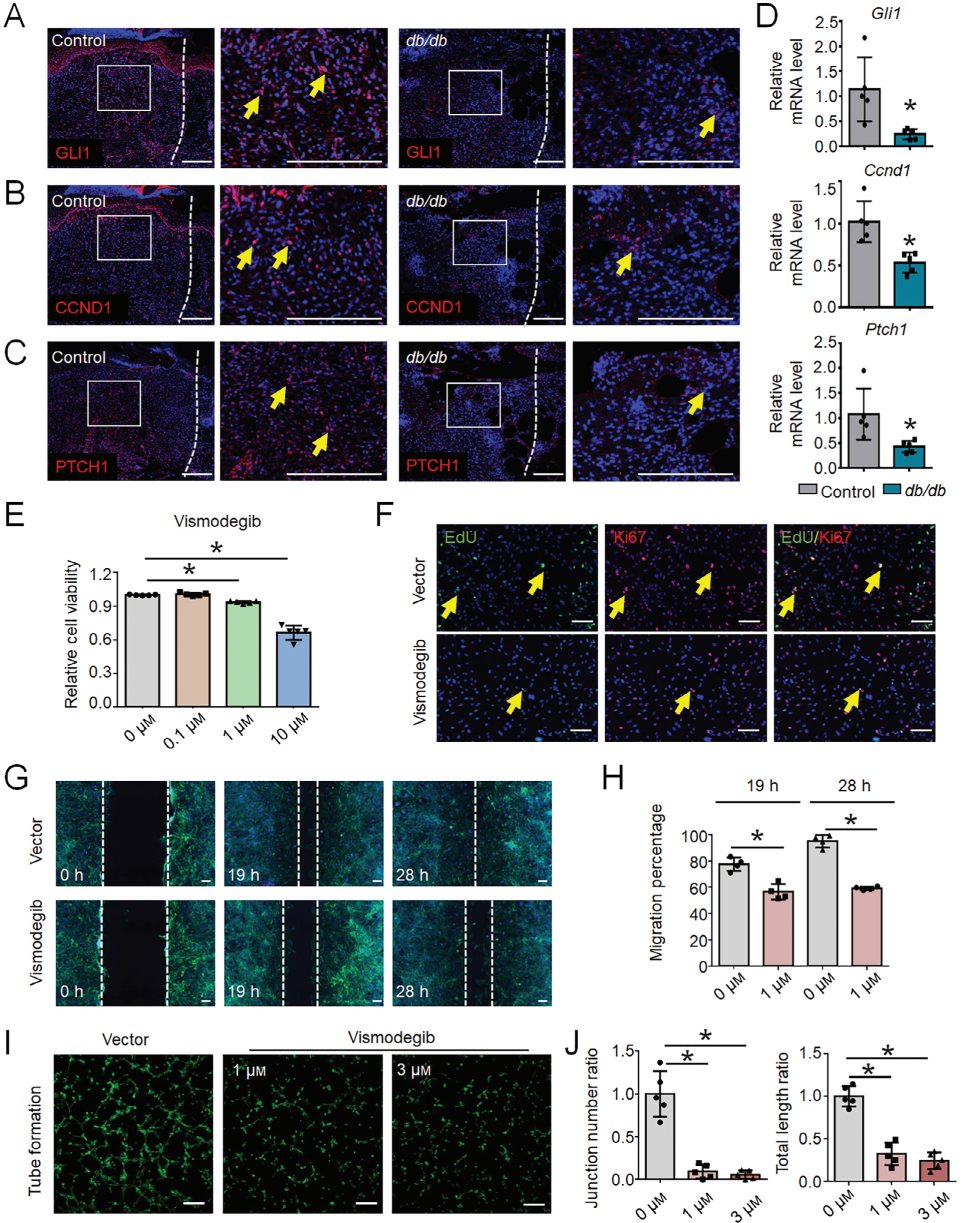
Downregulated Hedgehog signaling impairs dermal regeneration in DM. a‐c. Immunofluorescence staining for A) GLI1 (red), B) CCND1 (red), and C) PTCH1 (red) in the wounds of WT and *db/db* mice 9 days after surgery. White dashed lines mark the wound boundaries. Yellow arrows indicate positive signals. D) *Gli1, Ccnd1, and Ptch1* mRNA expression in the wounds of WT and *db/db* mice 3 days after surgery, as measured by qPCR. Data are presented as the mean ± SD, n = 5. P‐values were calculated using a two‐tailed Student's t‐test. *P < 0.05. E) Cell viability of dermal fibroblasts treated with vismodegib for 2 days, as determined using a Cell Counting Kit‐8 assay. Data are presented as the mean ± SD, n = 5. P‐values were calculated using ANOVA with Dunnett's multiple comparisons test. *P < 0.05. F) Immunofluorescence staining for Ki67 (red) and visualization of EdU (green) in dermal fibroblasts after treatment with 1 µm vismodegib or control vector for 24 h. Yellow arrows indicate proliferating cells. G) Representative images and H) quantification of the in vitro scratch assay results of dermal fibroblasts after treatment with 1 µm vismodegib or control vector for 0, 19, and 28 h. Dotted lines indicate the initial scratch edges. Data are presented as the mean ± SD, n = 4. P‐values were calculated using a two‐tailed Student's t‐test, *P < 0.05. I) Representative images and J) quantification of HUVEC tube formation after treatment with 1 µm vismodegib or control vector for 2 h. Data are presented as the mean ± SD, n = 5. P‐values were calculated using ANOVA with Dunnett's multiple comparisons test, *P < 0.05. Scale bar, 200 µm.

The Hh signaling pathway is one of most important signaling pathways regulating the development, repair, and maintenance of stem cells in the skin and appendages.^[^ ^20^, ^34^, ^35^, ^36^ ^]^ To investigate whether the impaired proliferation of dermal fibroblasts and vascularization of endothelial cells in DM are associated with downregulated Hh signaling, we initially evaluated the expression of GLI1, CCND1, and PTCH1, which are reader‐outs of Hh signaling pathways, in diabetic wounds. Using immunofluorescence staining (**Figure** **4**A–C) and quantitative reverse transcription PCR (qPCR) (Figure 4D), we found that Hh signaling was significantly downregulated during wound healing in DM mice compared with control mice. This suggests that the downregulation of Hh signaling during the wound‐healing process in DM may contribute to the impaired proliferation of dermal fibroblasts and the vascularization of endothelial cells. To test this hypothesis, we further evaluated the effect of the Hh signaling inhibitor vismodegib on fibroblast proliferation and migration using the CCK8 (Figure 4E), EdU and Ki67 immunofluorescence (Figure 4F), and scratch migration assays (Figure 4G,H). It indicated that the downregulation of Hh signaling inhibited dermal fibroblast proliferation and migration. A tube formation assay using HUVECs was also conducted to evaluate the function of Hh signaling in blood vessel formation. Downregulation of Hh signaling significantly reduced the junction number and branch length of HUVECs (Figure 4I,J). These results showed that suppressing the Hh signaling pathway using vismodegib inhibited fibroblast proliferation and migration, repressed angiogenesis, and impaired the wound repair process.

### 2.4 Hedgehog Signaling Activation Fails to Promote Regeneration Under Inflammatory Conditions

**Figure 5:**
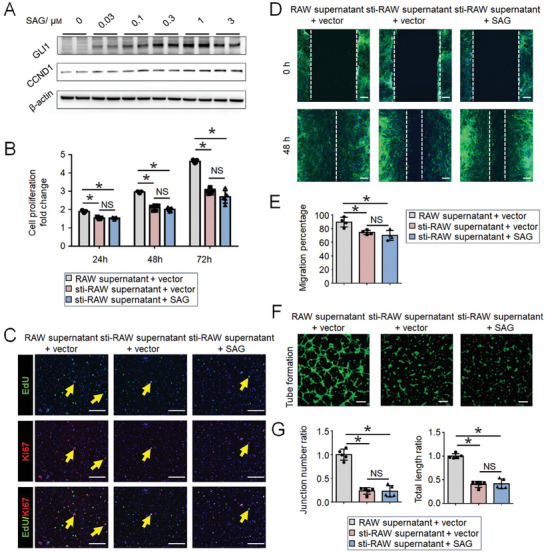
Hedgehog signaling activation fails to promote regeneration under inflammatory conditions. A) GLI1 and CCND1 protein expression in dermal fibroblasts treated with SAG or the control vector for 2 days. B) Cell proliferation fold change in dermal fibroblasts at 24, 48, and 72 h after co‐culture within RAW 264.7 cells supernatant with (sti‐RAW) or without LPS stimulation and supplemented with a vector or 1 µm SAG, as determined using a Cell Counting Kit‐8 assay. Data presented as the mean ± SD, n = 5 biologically independent samples. P‐values were calculated using ANOVA with Tukey's multiple comparisons test. *P < 0.05. NS, not significant, P > 0.05. C) Immunofluorescence staining for Ki67 (red) and visualization of EdU (green) in dermal fibroblasts after co‐culture within the supernatant of sti‐RAW 264.7 cells or RAW 264.7 cells without LPS stimulation supplemented with vector or 1 µm SAG for 48 h. D) Representative images and E) quantification of the results of the in vitro scratch assay of dermal fibroblasts co‐cultured within the supernatant of sti‐RAW 264.7 cells or RAW 264.7 cells without LPS stimulation supplemented with vector or 1 µm SAG for 48 h. Dotted lines indicate the initial scratch edges. Data are presented as the mean ± SD, n = 4. P‐values were calculated using ANOVA with Tukey's multiple comparisons test. *P < 0.05. NS, not significant, P > 0.05. F) Representative images and G) quantification of tube formation in HUVECs co‐cultured within the supernatant of sti‐RAW 264.7 cells or RAW 264.7 cells without LPS stimulation supplemented with vector or 1 µm SAG for 2 h. Data are presented as the mean ± SD, n = 5. P‐values were calculated using ANOVA with Tukey's multiple comparisons test, *P < 0.05. NS, not significant, P > 0.05. Scale bar, 200 µm.

To explore the therapeutic potential of the Hh signaling agonist SAG for wound healing in DM, we initially evaluated the effect of various concentrations of SAG on Hh signaling activation in dermal fibroblasts. We found that SAG could significantly activate Hh signaling in dermal fibroblasts at a concentration of 1 µm (**Figure** **5**A). Dermal fibroblasts were then co‐cultured within RAW 264.7 cells supernatant with or without LPS stimulation and supplemented with a vector or 1 µm SAG. Using the CCK8 (Figure 5B), EdU labeling and Ki67 immunofluorescence (Figure 5C), and scratch migration assays (Figure 5D,E), we found that the inflammatory supernatant inhibited the proliferation and migration of dermal fibroblasts. In addition, the inflammatory supernatant containing LPS‐stimulated macrophages inhibited blood vessel formation in HUVECs (Figure 5F,G). In addition, SAG failed to rescue the inflammation‐mediated suppression of the proliferation of dermal fibroblasts and vascularization.

Therefore, we conclude that impaired fibroblast proliferation, migration, and angiogenesis of endothelial cells in DM are associated with both Hh signaling downregulation and an excessive inflammatory response. Moreover, without a bona fide immune microenvironment, Hh signaling activation fails to effectively rescue the suppressed fibroblast proliferation and migration, as well as the vascularization of endothelial cells.

### 2.5 The Silk‐Based Core‐Shell MN Patch is Capable of Programmed Drug Release

To target the pathogenesis of delayed wound healing in DM, we hypothesized that the combination of a BRD9 degrader in the early inflammatory phase and SAG in the later regeneration phase could be an ideal therapy for effective skin regeneration in DM. Therefore, a silk‐based core‐shell MN patch was generated to modulate the immune microenvironment and regeneration process that matches the healing phases in DM.

**Figure 6:**
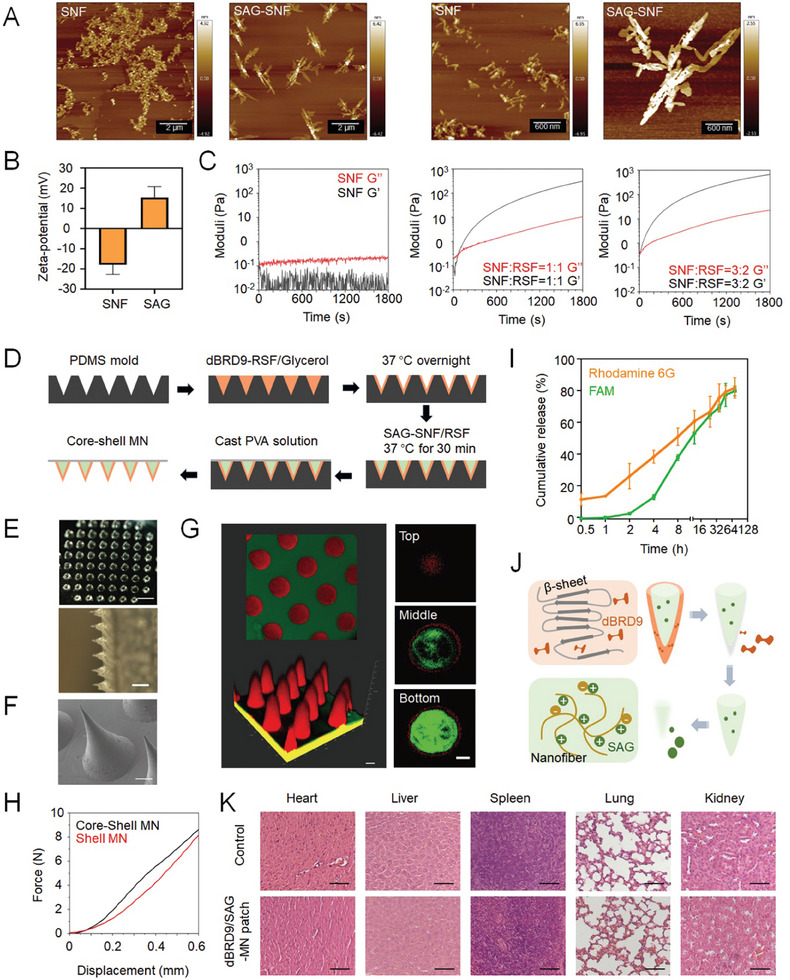
Fabrication and characterizations of the silk‐based core‐shell MN patch. A) AFM detection of the binding interaction between SAG and SNF. Scale bars: left panel, 2 µm; right panel, 600 nm. B) Zeta potential of SNF and SAG. C) Oscillatory time sweep analysis at a constant frequency of 1 Hz for SNF‐RSF hydrogel. D) Schematic diagram of the silk‐based core‐shell MN patch construction. E) Representative images of the silk‐based core‐shell MN patch. Scale bar, 1 mm. F) SEM observation of MN morphology. Scale bar, 100 µm. G) Confocal microscopy observation of the fluorescently labeled core‐shell MN patch (Rhodamine 6G‐labeled shell, FAM‐labeled core). Scale bar, 200 µm. H) Mechanical properties of the prepared MNs with core‐shell or shell structure. I) Cumulative release of model drugs from the core‐shell MN patch in vitro. *n* = 6. J) Schematic diagram of the gradient drug release capability of the core‐shell MNs. K) Tissue sections of the heart, liver, lungs, spleen, and kidneys of wild‐type mice treated with or without the silk‐based core‐shell MN patch for 10 days. Scale bar, 100 µm.

To construct silk‐based core‐shell MN patches, regenerated silk fibroin (RSF) and SNF are separately utilized. The SNF has the function of inducing rapid gelation of the regenerated silk protein,^[^ ^37^ ^]^ and their electronegativity can restrict early drug release through electrostatic adsorption.^[^ ^38^ ^]^ Then the electrostatic interaction between the negatively charged SNFs and positively charged SAG was evaluated using atomic force microscopy (AFM). The results revealed that the SNFs were in the form of short fibers after sonication. However, upon mixing with SAG, the sizes and widths of the SNFs significantly increased, suggesting that SAG may bind to SNFs through electrostatic interactions (**Figure** **6**A). We also measured the zeta potential of the SNF and SAG separately. The zeta potential of SNF was ≈−17.87 ± 4.81 mV, whereas the zeta potential of SAG was 15.37 ± 5.47 mV (Figure 6B), demonstrating that SNF can adsorb SAG through electrostatic interactions. These results were consistent with previous findings.^[^ ^39^, ^40^ ^]^ The choice of inner materials is crucial; they should exhibit initial fluidity and quickly form a gel after entering the MN interior to prevent the dissolution of the outer layer. Therefore, rheology tests were performed to measure the SNF‐triggered gelation process. The results demonstrated that as the proportion of SNF increased, the gelation time of the hydrogel significantly decreased (Figure 6C). Ultimately, we chose the mixture with an SNF to RSF ratio of 1:1, which had a gelation time within 5 minutes and was suitable for subsequent MN preparation.

According to the schematic diagram shown in Figure 6D, we fabricated a shell loaded with dBRD9 using RSF containing glycerol and a core loaded with SAG using negatively charged SNF. The representative photographs of the MN patches are shown in Figure 6E,F, demonstrating MNs with a height of 650 µm and a diameter of 350 µm. As illustrated in Figure 6B,C, the MNs are 650 µm in height and 350 µm in diameter. Then, confocal microscopy was used to observe the interface of the MN patches, which revealed a distinct core‐shell structure (Figure 6G). We further examined the mechanical strength of the prepared MN patches. Compression test revealed that the mechanical strength of the MN patch with an RSF shell is lower than that of the core‐shell structure. Adding the silk fibroin core enhanced the mechanical strength, making it more conducive for MNs to penetrate skin tissue (Figure 6H).

Furthermore, we assessed the gradient drug release capability of the core‐shell MNs. The results revealed that the drug loaded in the shell was rapidly released, ≈40% of which was released within the initial 4 h, while the drug loaded in the core released ≈15%, demonstrating a significant difference in release rates. After 72 h, the drug release rates from both layers reached ≈80% (Figure 6I). The gradient drug release is a combined result of multiple mechanisms, including silk protein degradation, free diffusion of drug molecules, physical barriers, and electrostatic interactions between molecules (Figure 6J).

The toxicity and safety of the silk‐based core‐shell MN patches were further evaluated in WT mice. After 10 days of treatment, the mice exhibited no obvious pathological changes in the vital organs, such as the heart, liver, spleen, lungs, and kidneys (Figure 6K).

### 2.6 The Programmed MN Patch Modulates the Wound Immune and Regeneration Processes, Matching the Healing Phases

**Figure 7:**
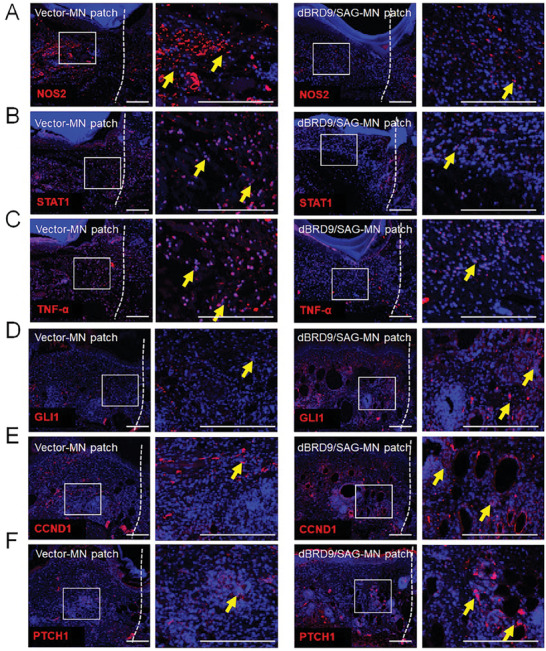
The silk‐based core‐shell MN patch inhibits excessive inflammation and activates Hedgehog signaling in a programmable manner. Immunofluorescence staining for A) NOS2 (red), B) STAT1 (red), and C) TNF‐*α* (red) in the wounds of the dBRD9/SAG‐loaded MNs group compared with the control group of *db/db* mice 3 days after surgery. Immunofluorescence staining for D) GLI1 (red), E) CCND1 (red), and F) PTCH1 (red) in the wounds of the control and dBRD9/SAG‐loaded MNs group of *db/db* mice 8 days after surgery. White dashed lines mark the wound boundary. Yellow arrows indicate positive signals. Scale bar, 200 µm.

We further assessed the therapeutic effects of the programmed MN patch on skin wounds in diabetic *db/db* mice in vivo. Using immunofluorescence staining of iNOS, STAT1, and TNF‐*α*, we detected an attenuation of the inflammatory response in the diabetic wound in the programmed dBRD9/SAG MN patch group compared with the blank‐loaded group at 3 days post‐surgery (**Figure** **7**A–C). This result indicated that the programmed MN patch could reduce excessive inflammation at the wound site in DM in the early phase. Eight days post‐surgery, immunofluorescence staining also revealed rescued Hh signaling activity with a higher expression of GLI1, CCND1, and PTCH1 at the wound site in the programmed dBRD9/SAG MN patch group compared to the control group (Figure 7D–F). It suggests that programmed MN patches dynamically modulate the wound immune microenvironment and the regeneration process to match the healing phases.

**Figure 8:**
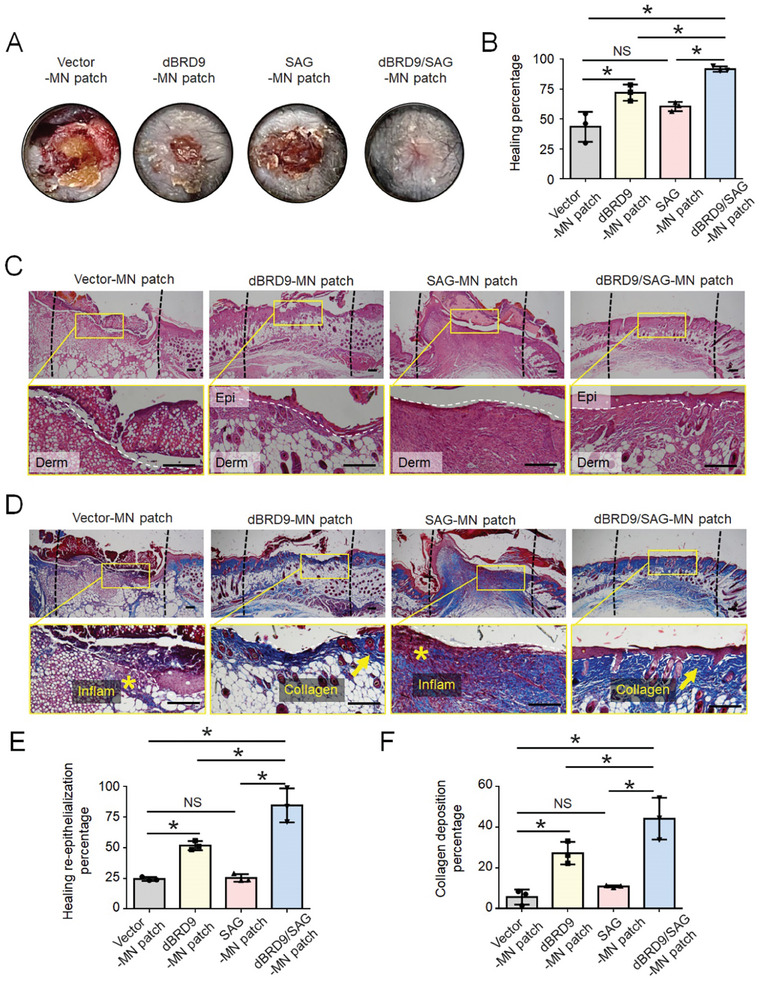
The silk‐based core‐shell MN patch promotes wound healing in HFD/STZ‐induced type‐2 diabetic mice. A) Representative image and B) quantification of the wound healing after treatment with dBRD9‐loaded MNs, SAG‐loaded MNs, and dBRD9/SAG‐loaded MNs compared with the control group of HFD/STZ‐induced type 2 diabetic mice 8 days after surgery. Data are presented as the mean ± SD, n = 3. P‐values were calculated using ANOVA with Tukey's multiple comparisons test. *P < 0.05. NS, not significant, P > 0.05. C) Representative H&E and D) Masson's trichrome staining images of wound healing after treatment with dBRD9‐loaded MNs, SAG‐loaded MNs, and dBRD9/SAG‐loaded MNs compared with the control group of HFD/STZ‐induced type‐2 diabetic mice 8 days after surgery. Black dashed lines mark the wound boundary. White dashed lines indicate the dermis. The yellow arrow in D) indicates the collagen bundles. The asterisks indicate infiltrating inflammatory cells. Epi, epidermis; derm, dermis; inflam, infiltrating inflammatory cell. Quantification of E) re‐epithelialization percentage and F) collagen deposition percentage after treatment with dBRD9‐loaded MNs, SAG‐loaded MNs, and dBRD9/SAG‐loaded MNs compared with the control group of HFD/STZ‐induced type 2 diabetic mice 8 days after surgery. Data are presented as the mean ± SD, n = 3. P‐values were calculated using ANOVA with Tukey's multiple comparisons test. *P < 0.05. NS, not significant, P > 0.05. Scale bar, 200 µm.

To further analyze the wound healing effect of the silk‐based core‐shell MN patch in DM, we utilized it in a high‐fat diet/streptozotocin (HFD/STZ) induced type 2 DM model. Statistical analysis of the wound healing coverage showed that the programmed dBRD9/SAG MN patch promoted re‐epithelialization, indicating a better healing effect than the blank‐loaded or single‐drug‐loaded MN patch (**Figure** **8**A,B). Although the dBRD9‐loaded MN patch exhibited a significantly better healing effect than the blank vector‐loaded MN patch, the SAG‐loaded MN patch exhibited no apparent effect on wound healing in DM, which is consistent with the above finding in vitro that without a bona fide immune microenvironment, Hh signaling activation alone fails to effectively rescue the suppressed proliferation ability of dermal fibroblasts and vascularization of endothelial cells. Healing and collagen deposition in each group of wounds were compared using H&E (Figure 8C) and Masson's staining (Figure 8D) on histological sections. Although the dBRD9‐loaded MN patch exhibited anti‐inflammatory effects on the wound compared to the blank vector‐loaded MN patch, inflammatory cell infiltration was still severe in the single SAG‐loaded MN group. We found that the programmed dBRD9/SAG‐loaded MN patch promoted re‐epithelialization (Figure 8E) and collagen deposition (Figure 8F) compared to the blank vector‐loaded or single drug‐loaded MN patches.

## 3 Discussion

The skin is the largest human organ in terms of area and weight. It is the primary physical barrier protecting the body from the external environment, such as microorganisms, dehydration, mechanical damage, and UV light radiation. Wound healing is the physiological restorative process of the skin barrier after trauma.^[^ ^41^ ^]^ This process includes successive but overlapping phases, including the inflammatory, proliferative, and remodeling phases.^[^ ^42^ ^]^ The effective suppression and transition of the early inflammatory phase to the later regeneration phase is a critical event during wound healing.^[^ ^43^ ^]^ Although tremendous efforts have been made to identify an intervention for diabetic wound healing, effective translation from basic research into clinical practice remains challenging.^[^ ^44^, ^45^ ^]^ To date, FDA‐approved treatments for diabetic wound are barely satisfactory, despite showing significant efficacy in clinical trials.^[^ ^46^ ^]^ Therefore, in‐depth knowledge of the cellular and molecular pathogenesis of delayed wound healing in DM in a particular context and targeted therapy might be a prerequisite for overcoming the significant bottleneck in translating interventions for wound healing in DM.

Unlike genetic modulation, epigenetic therapy can reversibly modulate the expression of disease‐related genes without altering their normal DNA sequences. Rapid advances in our understanding of the epigenetic regulation of DM have led to the development of many new epigenetic therapeutic drugs.^[^ ^47^ ^]^ These epigenetic targets open the possibility of treating DM and preventing its complications. For example, inhibiting the methylation of *Sod2* and *Mmp9* has shown some success in reducing the symptoms of diabetic retinopathy.^[^ ^48^ ^]^ Apabetalone, an oral bromodomain and extraterminal (BET) inhibitor, exhibits both anti‐inflammatory properties and the ability to reduce alkaline phosphatase levels in people with a probability of type 2 DM‐associated cardiovascular and chronic kidney disease.^[^ ^49^ ^]^

Several studies have shown promising strategies for diabetic wound healing based on DNA methylation, histone lysine methylation/demethylation, and noncoding RNA.^[^ ^50^, ^51^, ^52^, ^53^, ^54^ ^]^ For example, it has been reported that DNA methyltransferase 1 (DNMT1) induces DNA methylation and suppression of Notch1, PU.1, and Klf4 in hematopoietic stem cells and skews pro‐inflammatory polarization of macrophages. Targeting DNMT1 in macrophages improved wound healing in a T2DM mouse model.^[^ ^50^, ^51^ ^]^ Another study has shown that increased histone demethylase Jumonji domain‐containing protein D3 (JMJD3) expression increased STING and inflammatory response in diabetic wounds. A corresponding therapeutic strategy for macrophage‐targeted JMJD3 suppression promotes diabetic wound healing by ameliorating persistent inflammation.^[^ ^53^, ^54^, ^55^ ^]^ Although ATP‐dependent chromatin remodeling has been demonstrated to be critical for metabolic disease, there is a paucity of these regulatory functions in diabetes or wound healing.^[^ ^56^ ^]^

BRD9 has been identified as a critical subunit of the ncBAF complex. A recent study in mouse models has shown that the pharmacological inhibition of BRD9 restores *β*‐cell function and ameliorates hyperglycemia through a ligand‐dependent switch between the vitamin D receptor and BAF remodeling complexes.^[^ ^57^ ^]^ Besides, BRD9 inhibition limits inflammation by blocking the induction of ISG expression.^[^ ^15^, ^16^, ^17^ ^]^ However, whether the local application of BRD9 degraders or inhibitors could attenuate the sustained inflammatory state of the wound in diabetes remains to be elucidated.

Using a reindeer antler skin model, it has been reported that pathologic fibroblast‐immune interactions in wound healing diminish the regenerative ability of the skin.^[^ ^58^ ^]^ Besides sustained chronic inflammation, impeded fibroblast proliferation and vascularization of endothelial cells during the later phases also result in impaired wound healing ability in diabetes.^[^ ^19^ ^]^ In the present study, we found that without a bona fide immune microenvironment, Hh signaling activation failed to effectively rescue the suppressed fibroblast proliferation and migration and the vascularization of endothelial cells. These findings suggest that sequential and multi‐target therapies are promising strategies for wound healing in DM.

To achieve programmed drug delivery, we propose a silk‐based core‐shell MN construction strategy. Initially, we employed the polyol‐induced formation of *β*‐sheets within the silk protein shell to enhance the mechanical strength and toughness while avoiding the use of toxic substances such as methanol. The formed *β*‐sheet nanocrystals during silk fibroin assembly, on one hand, enhance the cross‐linking between silk fibroin molecules, and on the other hand, owing to their nanoscale nature, in conjunction with uniform shear deformation that efficiently harnesses hydrogen bonds and the occurrence of dissipative molecular stick‐slip deformation, contribute significantly to the enhancement of mechanical properties.^[^ ^59^, ^60^ ^]^ Next, we applied the rapid *β*‐sheet‐inducing properties of nanoscale silk fibroin fibers to construct the MN core, preventing the dissolution of the shell's silk protein. Moreover, the electronegativity of nanoscale silk fibroin molecules allows for the adsorption of positively charged drugs, effectively achieving the sustained release of SAG. This eco‐friendly MN fabrication method avoids introducing other chemical components and demonstrates good gradient‐controlled release effects.

Therefore, this study could potentially contribute much not only to the literature on the use of natural biomaterials as starting materials for core‐shell‐structured MN technology but also to the clinical management of wound healing and skin regeneration in patients with DM. Despite the promising effect of silk‐based core‐shell MNs on wound healing in a diabetic mouse model, several limitations still need to be acknowledged. Unlike the acute wound model on the dorsal skin of mice used in this study, diabetic foot ulcers, the most common complication with the highest morbidity of lower‐extremity amputation in patients with diabetes, are confronted with more complex challenges such as recurrent infections, granulation tissue formation, and excessive pressure on the ulcer site.^[^ ^61^ ^]^ Therefore, the therapeutic efficacy of MNs for diabetic foot ulcers must be further evaluated and improved. In addition, more versatile and intelligent MN systems with preventive, blood glucose monitoring and regulation, and environment‐responsive functions are promising for a broader range of applications and are expected to bring better social and economic benefits.

## 4 Conclusion

In summary, we developed a pure silk fibroin core‐shell MN fabrication strategy to dynamically modulate the wound immune microenvironment and regeneration process, matching the healing phases of DM. The shells of the MNs mitigated overt inflammatory processes by releasing the epigenetic drug dBRD9 in the early phase. Subsequently, the positively charged SAG is released from the negatively charged inner SNF, promoting a phase transition from inflammation to regeneration, re‐epithelialization, collagen deposition, and angiogenesis. These findings suggest that the programmed silk‐based core‐shell MN array patch is an ideal therapeutic strategy for effective functional skin regeneration in DM.

## 5 Experimental Section

### Mice

Type 2 diabetic mice (*BKS‐Lepr^em2Cd479/Nju^*, *Lepr^db/db^*, *db/db*; strain NO. T002407) and age‐matched WT mice were obtained from GemPharmatech (Nanjing, China). For type 2 DM induced by a high‐fat diet/streptozotocin (HFD/STZ), mice were fed an HFD (60% of calories from fat) from 4 weeks of age. At 10 weeks of age, HFD‐fed mice were intraperitoneally injected with an STZ solution (40 mg K‐1 G‐1; Yeasen, Shanghai, China) to type 2 DM. The animals were kept in a pathogen‐free environment with stable temperature and humidity.^[^ ^62^ ^]^ All procedures were approved by the Institutional Animal Care and Use Committee of the Ninth People's Hospital, School of Medicine, Shanghai Jiao Tong University (SH9H‐2022‐A926‐1).

### Cell Culture

Dermal fibroblasts were isolated from newborn mice. After removing fat and other membranous tissues, the dissected trunk skin was cut into small pieces, digested, and cultured in DMEM supplemented with 10% fetal bovine serum (FBS; OriCell FBSAD‐01011‐500, Cyagen Biosciences, Guangzhou, China). Dermal passages 2 to 4 were used for further experiments.^[^ ^63^ ^]^

Murine mononuclear RAW 264.7 line was cultured in DMEM supplemented with 10% FBS. HUVECs were grown in Endothelial Cell Medium (ScienCell, Carlsbad, CA, USA). For macrophage activation, RAW 264.7 cells were cultured in complete culture medium supplemented with 100 ng mL^−1^ LPS.

For the drug treatment experiments, the cells were treated with dBRD9 (R&D Systems, 6606), vismodegib (Selleck, S1082), or SAG (Selleck, S7779) at the indicated dose 24 h after plating. For the co‐culture assay, RAW 264.7 macrophage cells were activated with LPS or vector for 2 days. Then the supernatant was collected, centrifuged, and filtered using a 0.2 µm filter for further co‐culture assays.^[^ ^64^ ^]^ Next, dermal fibroblasts were cultured with the RAW 264.7 cell supernatant with or without LPS stimulation, and supplemented with a vector or 1 µm SAG for functional assay evaluation.

### Preparation of a Regenerated Silk Fibroin (RSF) Solution

Silk cocoons were purchased from Lirui Biotechnology Co., Ltd. (Shanghai, China). Sodium carbonate (Na_2_CO_3_) and lithium bromide (LiBr) were obtained from Sangon Biotech (Shanghai, China). Chopped silk cocoons (5 g) were boiled in a Na_2_CO_3_ solution (2 L, 0.02 m) for 30 min. The extracted silk fibroin was rinsed three times with distilled water. Then, 1 g of silk fibroin was added to 4 mL of a 9.3 m LiBr solution in a 60 °C water bath until the silk fibroin was fully dissolved. This was followed by dialysis against deionized water for two days. The post‐dialysis protein solution was centrifuged and stored at 4 °C for subsequent use.

### Preparation of Silk Fibroin Nanofibers (SNFs)

As previously reported, the preparation of SNF solutions involved rapid and gradual concentration steps, followed by dilution with distilled water and incubation at controlled temperatures to yield nanofibers with high crystallinity.^[^ ^65^ ^]^ The procedure is detailed as follows. The RSF solution, prepared beforehand, was transferred to a beaker and rapidly concentrated by heating in a 60 °C oven for 24 h. Subsequently, the solution underwent gradual concentration in a fume hood over 3 days, resulting in a solution with an approximate concentration of 25 wt.% silk fibroin, which was then allowed to stabilize for 2 days in a refrigerator at 4 °C. The concentrated silk fibroin solution was then diluted to 2 wt.% using ultrapure water and thoroughly mixed via magnetic stirring. The solution was sealed and incubated at 60 °C. The generated SNF solution was subsequently stored at 4 °C for future applications.

### Programmed MN Patch Construction and Characterization

Core‐shell silk fibroin MNs were prepared by adding glycerol (Sangon Biotech) to the solution to achieve an RSF‐to‐glycerol ratio of 1:0.4. After thorough mixing with 100 µm dBRD9 or Rhodamine 6G (Topscience, Shanghai, China), 50 µL solution was transferred into a mold and centrifuged at 4500 rpm for 5 min. After aspiration, the mold was incubated at 37 °C for 4 h. Next, the SNF hydrogel was sonicated to form a solution, filtered for sterilization, and 200 µm SAG or FAM (Topscience, Shanghai, China) was added. This solution was mixed with the RSF solution in a 1:1 ratio, centrifuged in the mold mentioned above at 4500 rpm for 5 min. After incubated at 37 °C for 30 min, the mold was added with 20% polyvinyl alcohol (PVA; Sangon Biotech) and incubated overnight at room temperature. The morphology of the fabricated core‐shell MNs was examined using optical microscopy, scanning electron microscopy (Nova NanoSEM 230, FEI, US), and confocal laser scanning microscopy (Leica TCS SP8).

The SNF solution was diluted to a concentration of 0.5 wt.%. Next, 500 µL of the above solution was added with 2 mg of SAG powder. The mixture was shaken at 4 °C for 2–4 h, then diluted 1:100. A small amount was dropped onto a mica sheet, followed by infrared drying. A solution without adding SAG powder was used as a control. The morphology of the samples was observed at room temperature using an atomic force microscope.

### Zeta Potential Measurement

Zeta potential measurements were carried out at 25 °C using a computerized Nano Z3000 Zetasizer (Nicomp, US).

### Rheological Monitoring of Gelation

The SNF‐triggered gelation process was performed using a HAAKE MARS rheometer (Thermo, US) with a 60 mm parallel‐plate configuration. A 1 mL aliquot solution with different SNF proportions was immediately transferred onto the Peltier plate. Time sweeps were carried out at 25 °C with a frequency of 1 Hz and a strain of 1%, which was within the linear viscoelastic range.

### Compression Test of MN Patches

Mechanical properties of core‐shell or shell MN were measured using a universal testing system (HY‐0230, Hengyi, China). The MN patches were placed on the plate, and the sensor approaching rate was 3 mm min^−1^. The process of measuring the compression force commenced upon the sensor's contact with the needle tips and concluded when the sensor had advanced an additional 0.6 mm vertically.

### Drug Release Evaluation

To evaluate the drug release profiles of the constructed core‐shell MNs, Rhodamine 6G and FAM were used as model molecules. In brief, 5 µg of Rhodamine 6G and FAM were separately incorporated into the inner and outer layers. Subsequently, the MNs were submerged in phosphate‐buffered saline (PBS) (100 mg: 5 mL) at 37 °C. 1 mL of PBS was withdrawn for drug release detection at each time point. The concentrations of Rhodamine 6G and FAM were quantified experimentally using a standard calibration curve. The fluorescence of Rhodamine 6G (excitation: 580 nm, emission: 620 nm) and FAM (excitation: 480 nm, emission: 535 nm) were detected using a microplate reader (SPARK, TECAN, Switzerland).

### Cell Viability and Proliferation

To evaluate the cytotoxicity of the constructed MN patches, the patches were soaked in a complete culture medium (80 mg: 4 mL) for 48 h before the supernatant was collected. Dermal fibroblasts and HUVECs were cultured in the solutions extracted from the vector‐loaded and dBRD9‐loaded MN patch for 24 h, and a blank solution was used as the control. The viability of dermal fibroblasts and HUVECs was assessed using the CCK‐8 assay (Dojindo Kagaku, Japan). EdU incorporation and detection (Thermo Fisher, C10637 was conducted by following the manufacturer's instructions.

### Cell Migration Assay

Briefly, dermal fibroblasts were seeded and cultured until they reached confluence. After serum starvation for 24 h, scratches were made on the culture vessels using 1000‐µL pipette tips. After washing with PBS twice to remove the dislodged cells, different culture media supplemented with either vehicle, varying concentrations of vismodegib, or supernatants from RAW 264.7 macrophage cells after LPS‐ or non‐LPS‐stimulation supplemented with vector or 1 µm SAG were added. Cell migration was observed and recorded using a Microscope.

### Tube Formation Assay

As previously described,^[^ ^66^, ^67^ ^]^ GF‐reduced Matrigel (BD Biosciences, USA) was incubated in cell culture plates at 37 °C for gelation. HUVECs were seeded onto the gel containing different culture media supplemented with either vehicle or varying concentrations of vismodegib or supernatant from RAW 264.7 macrophage cells after LPS‐ or non‐LPS‐stimulation supplemented with vector or 1 µm SAG. After incubating under 37 °C for 2 h, cells were fixed and stained with a fluorescein isothiocyanate (FITC)‐phalloidin‐PBS solution (Yeasen, Shanghai, China). Fluorescence images of five randomly selected fields were obtained using a Leica TCS SP8 Confocal Microscope.

### Wound Healing In Vivo

To evaluate wound healing in vivo, 6‐mm full‐thickness wound defects were constructed in type 2 diabetic mice. After removing the fur, a sterile 6‐mm biopsy punch was used to mark two circular wound areas at the shoulder level. The marked circular regions were excised to construct a full‐thickness wound. Next, the MN patches were immediately applied to the full‐thickness wound, the wounds were fixed with a silicone splint, and then covered with a gauze pad.^[^ ^68^ ^]^ Finally, the wound areas were quantified using ImageJ software with photos taken at 0 and 8 days post‐surgery.

### Histological Analysis of Wound Tissues

Wound tissues were fixed, dehydrated, and sectioned as previously described.^[^ ^17^, ^62^ ^]^ H&E (Servicebio, G1005, Wuhan, China) and Masson's trichrome (Leagene, DC0033, Beijing, China) staining were conducted by following instructions. The width and thickness of the newly formed epithelium and collagen deposition were used as objective measures to evaluate wound healing.

### Immunofluorescence Staining

Immunofluorescence staining and image capture were conducted as previously described.^[^ ^17^, ^62^ ^]^ The primary antibodies are as follows: PECAM‐1 (Abcam, ab281583, 1:100), KRT14 (ABclonal, A19039, 1:100), NOS2 (Abcam, ab178945, 1:100), STAT1 (Affinity biosciences, AF6300, 1:100), TNF‐*α* (Affinity biosciences, AF7014, 1:100), GLI1 (Novus, NBP1‐78259, 1:100), PTCH1 (ABclonal, A14772, 1:100), CCND1 (Abcam, ab16663, 1:100), Ki‐67 (Cell signaling, 9129, 1:100). The secondary antibodies used were: Alexa Fluor 594‐conjugated donkey anti‐rabbit IgG (Thermo Fisher Scientific, A‐21207, 1:200), Alexa Fluor 488‐conjugated goat anti‐rabbit IgG (Thermo Fisher Scientific, A‐11008, 1:200), Alexa Fluor 594 ‐conjugated goat anti‐mouse IgG (Thermo Fisher Scientific, A‐11005, 1:200), and Alexa Fluor 488‐conjugated goat anti‐mouse IgG (Thermo Fisher Scientific, A‐11001, 1:200).

### Quantitative Reverse Transcription PCR (qPCR)

RNA was extracted and quantified as previously described.^[^ ^17^, ^62^ ^]^ Details of the primers used are listed in Table S1 (Supporting Information).

### Western Blot

Western blotting was performed as previously described.^[^ ^17^, ^62^ ^]^ The antibodies utilized for western blot were: GLI1 (Novus, NBP1‐78259, 1:1000), CCND1 (Abcam, ab16663, 1:1000), *β*‐actin (Abcam, ab20272HRP, 1:5000), and HRP‐conjugated secondary antibody (R&D, HAF008, 1:1000).

### Statistical Analysis

All statistical analyses were conducted using GraphPad Prism v6.01 software. The results were presented as the mean ± standard deviation (SD). For comparisons between the two groups, an unpaired two‐tailed t‐test was used. For comparisons between multiple groups, one‐way analysis of variance (ANOVA) with Tukey's or Dunnett's post‐hoc test was used. Statistical significance was defined as a probability (P) value < 0.05. The sample sizes (n), P‐values, and specific statistical tests performed for each experiment are described in the corresponding figure legends.

## Conflict of Interest

The authors declare no conflict of interest.

## Author Contributions

J.D. and X.J. conceived and designed the study. Y.L., M.Z., J.D., J.S., E.Y, J. X., G.Y, X.W., and L.X. performed the experiments. J.D. and M.Z. provided critical resources. J.D. and X.J. provided technical assistance and supervision. All authors contributed to the interpretation of experiments. J.D., Y.L., M.Z., and X.J. wrote the manuscript with input from all co‐authors. All authors discussed the results and contributed to the manuscript.

## Supporting information



Supporting Information

## Data Availability

The data that support the findings of this study are available from the corresponding author upon reasonable request.
