# Supplementary material for: Programmed BRD9 Degradation and Hedgehog Signaling Activation via Silk‐Based Core‐Shell Microneedles Promote Diabetic Wound Healing
Source: Adv Sci (Weinh). 2024 Oct 16;11(45):2404130. doi: 10.1002/advs.202404130 (PMC11615742; doi:10.1002/advs.202404130)
Supplement: Supplementary file 1 — Supporting Information [file ADVS-11-2404130-s001.pdf]

ADVANCED SCIENCE
Open Access

Supporting Information

for *Adv. Sci.*, DOI 10.1002/advs.202404130

Programmed BRD9 Degradation and Hedgehog Signaling Activation via Silk-Based Core-Shell Microneedles Promote Diabetic Wound Healing

Yili Liu, Mingliang Zhou, Jinrui Sun, Enhui Yao, Jingyi Xu, Guangzheng Yang, Xiaolin Wu, Ling Xu, Jiahui Du and Xinquan Jiang**

Supporting Information

Programmed BRD9 Degradation and Hedgehog Signaling Activation via Silk-Based Core-Shell Microneedles Promote Diabetic Wound Healing

Yili Liu, Mingliang Zhou, Jinrui Sun, Enhui Yao, Jingyi Xu, Guangzheng Yang, Xiaolin Wu, Ling Xu, Jiahui Du and Xinquan Jiang**

Table S1: List of primer sequences used for q-PCR.

Gene	Forward	Reverse
β-actin	GGCTGTATTCCCCTCCATCG	CCAGTTGGTAACAATGCCATGT
Nos2	GTTCTCAGCCCAACAATACAAGA	GTGGACGGGTCGATGTCAC
Tnf-α	CCCTCACACTCAGATCATCTTCT	GCTACGACGTGGGCTACAG
Il-1β	GCAACTGTTCCTGAACTCAACT	ATCTTTTGGGGTCCGTCAACT
Il-12α	CTGTGCCTTGGTAGCATCTATG	GCAGAGTCTCGCCATTATGATTC
Gli1	CCAAGCCAACTTTATGTCAGGG	AGCCCGCTTCTTTGTTAATTTGA
Ccnd1	GCGTACCCTGACACCAATCTC	CTCCTCTTCGCACTTCTGCTC
Ptch1	AAAGAACTGCGGCAAGTTTTTG	CTTCTCCTATCTTCTGACGGGT
